# Effects of Bilayer Nanofibrous Scaffolds Containing Curcumin/Lithospermi Radix Extract on Wound Healing in Streptozotocin-Induced Diabetic Rats

**DOI:** 10.3390/polym11111745

**Published:** 2019-10-24

**Authors:** Bo-Yin Yang, Chung-Hsuan Hu, Wei-Chien Huang, Chien-Yi Ho, Chun-Hsu Yao, Chiung-Hua Huang

**Affiliations:** 1 Graduate Institute of Chinese Medicine, School of Chinese Medicine, College of Chinese Medicine, China Medical University, Taichung 40402, Taiwan; u100058857@cmu.edu.tw; 2 City Beauty Health Consultant Ltd. Company, Taichung 40356, Taiwan; citybeauty168@gmail.com; 3 Graduate Institute of Biomedical Science, China Medical University, Taichung 40402, Taiwan; whuang@mail.cmu.edu.tw; 4 Department of Biomedical Imaging and Radiological Science, China Medical University, Taichung 40402, Taiwan; samsam172@yahoo.com.tw; 5 Biomaterials Translational Research Center, China Medical University Hospital, Taichung 40402, Taiwan; 6 Department of Biomedical Informatics, Asia University, Taichung 41354, Taiwan; 7 Department of Medical Laboratory Science and Biotechnology, Central Taiwan University of Science and Technology, Taichung 40601, Taiwan

**Keywords:** diabetic, curcumin, Lithospermi radix (LR), wound healing, TGF-*β*, IL-6, TNF-*α*

## Abstract

Impaired growth factor production, angiogenic response, macrophage function, and collagen accumulation have been shown to delay wound healing. Delayed wound healing is a debilitating complication of diabetes that leads to significant morbidity. In this study, curcumin and Lithospermi radix (LR) extract, which are used in traditional Chinese herbal medicine, were added within nanofibrous membranes to improve wound healing in a streptozotocin (STZ)-induced diabetic rat model. Gelatin-based nanofibers, which were constructed with curcumin and LR extract at a flow rate of 0.1 mL/hour and an applied voltage of 20 kV, were electrospun onto chitosan scaffolds to produce bilayer nanofibrous scaffolds (GC/L/C). The wounds treated with GC/L/C exhibited a higher recovery rate and transforming growth factor-beta (TGF-*β*) expression in Western blot assays. The decreased levels of pro-inflammatory markers, interleukin-6 (IL-6) and tumor necrosis factor-*α* (TNF-*α*), provided evidence for the anti-inflammatory effects of GC/L/C treatment. Chronic wounds treated with GC/L/C achieved better performance with a 58 ± 7% increase in recovery rate on the seventh day. Based on its anti-inflammatory and wound-healing effects, the GC/L/C bilayer nanofibrous scaffolds can be potential materials for chronic wound treatment.

## 1. Introduction

Wound healing is a complex event, requiring the interactions of many cell types, including inflammatory cells, fibroblasts, keratinocytes, and endothelial cells, as well as the involvement of growth factors and enzymes [1,2]. The progress of the healing process is impaired in chronic wounds, including those due to diabetes. Impaired growth factor production, angiogenic response, macrophage function, and collagen accumulation have been shown to delay the wound healing process [3,4]. Delayed wound healing is a major clinical problem for patients with diabetes. There are various topical dressings for treating the diabetic wound, including hydrocolloids, hydrogels, alginates, foam, and drug impregnated dressings.

The ideal biomaterial should mimic the characteristics of the desired in vivo environments for tissue engineering. In recent years, natural materials have drawn attention in their application as membranes for wound healing. Gelatin is a partially degraded product of collagen, and the most abundant protein in the extracellular matrix (ECM). Gelatin nanofiber, manufactured by an electrospinning technique, is a biodegradable membrane-forming material with considerable properties, including small pores, a high surface area, physical stability, and promotes cell adhesion, differentiation, and proliferation [5]. Its distinguishing features make the gelatin nanofiber an ideal material for wound dressing. The dried gelatin nanofibrous membrane becomes a thin layer and that can sometimes easily roll-up. Thus, an additional basal layer might provide structural support for gelatin nanofibers. Chitosan is a partially deacetylated product made from chitin and is a biocompatible and biodegradable polymer. It is widely used in wound dressing due to its many advantages like accelerating tissue regeneration, biocompatibility, and antibacterial property [6,7,8]. The amino groups of chitosan can electrostatically interact with –COO^−^ of gelatin [9]. Therefore, the chitosan scaffold might act as the basal layer to support the structure of the gelatin nanofibrous membrane.

Gelatin nanofibers have also been studied as potential drug carriers due to their large surface area and high porosity. Pharmaceutical ingredients which are commonly delivered using electrospun membranes include antibiotics, anti-inflammatory drugs, proteins, nucleic acids, and herbal extracts [10,11]. Lithospermi radix (LR) has been used as a traditional therapy for burns and skin diseases such as eczema and psoriasis [12]. Recent studies showed that LR has multiple pharmacological activities, including wound-healing, antibacterial, and anti-inflammatory effects. The active ingredients of LR contain *β*-hydroxy-isovaleryl-shikonin, shikonin, quinones, isobutyl-shikonin, and *α*-methyl-n-butyl-shikonin [13]. The beneficial effect of shikonin on skin wound healing is mainly related to the epithelial–mesenchymal transition (EMT), which is a cell transdifferentiation process that is important for wound-healing activity in human skin [14]. Evidence also showed that increased collagen synthesis and TGF-*β* expression in the LR-treated fibroblasts [15,16,17]. Another herbal component, curcumin, was incorporated into gelatin nanofibers. Curcumin, a natural polyphenol, is an active agent derived from plant *Curcuma longa* (turmeric). It has been shown to have a wide range of pharmacological activities, including anti-inflammatory, anti-oxidant, anti-bacterial effects, and wound healing effects [18,19]. LR and curcumin obtain different medical functions; the combination of two herbal components might provide a synergistic effect in wound healing.

In the wound healing process, several cytokines have been reported to play an essential role in the inflammatory reaction. Evidence has shown that pro-inflammatory cytokines (IL-6 and TNF-*α*) are involved in the up-regulation of inflammatory responses [20]. The decreased production of IL-6 and TNF-*α* indicates the suppression of the inflammatory response. TGF-*β*, a family of growth factors, exerts pleiotropic effects on wound healing by regulating cell proliferation, differentiation, extracellular matrix production, and modulating the immune response. An effective treatment could help cells precisely react to inflammatory mediators, growth factors, and cytokines, which are essential for effective wound recovery [21,22].

In this study, bilayer nanofibrous scaffolds were manufactured by electrospinning gelatin nanofibers onto chitosan scaffolds. The properties of the bilayer scaffold were characterized through physicochemical and in vivo wound healing analysis. The benefits of the bilayer scaffolds containing curcumin and LR extract on delayed wound healing in streptozotocin-diabetic rats were investigated [23]. The aim was to develop a potential material for chronic wound healing applications.

## 2. Materials and Methods

Poly-(vinyl) alcohol (PVA) (MW = 1400) was purchased from SHOWA (Tokyo, Japan). Chitosan (>75% deacetylated), glutaraldehyde, DAPI, and curcumin were acquired by Sigma (St. Louis, MO, USA). Mouse fibroblasts (L929) were cultured in Dulbecco’s modified Eagle’s medium (DMEM) supplemented with 10% fetal bovine serum (FBS). The reagents for cell culture were supplied from Invitrogen (Carlsbad, CA, USA).

### 2.1. Biochemical Analysis of Curcumin

#### 2.1.1. Cell Viability Assay

L929 cells were cultured at 1 × 104 cells/well in a 96 well-culture dish. The cell viability was performed by MTT (3-(4,5-dimethylthiazol-2-yl)-2,5-diphenyltetrazolium bromide) assay. Add MTT made up in the medium to a final concentration of 0.5 mg/mL. The microplate was incubated at 37 °C for 4 h. After removing the medium from the microplate, the solubilizing solution was added to each well. After shaking for 3 min, the optical density was read at a wavelength of 570 nm using an ELISA reader (Molecular Devices, San Jose, CA, USA).

#### 2.1.2. Cell Migration Assay

L929 cell was planted at 8 × 10^4^ cell/well in the 12 well-culture dishes and using a tip to draw a line in the middle of culture dish after 24 h incubation. Each well was washed with PBS buffer for 3 times and added 9:1 culture medium with different concentrations of curcumin. After incubation for 48 h, L929 cells were stained with Liu’s stain and observed using an inverted microscope (Axiovert, 25).

#### 2.1.3. Tyrosinase Inhibition Assay

Tyrosinase inhibitory assay was performed with l-3,4-dihydroxyphenylalanine (L-DOPA) as a substrate. The inhibition activity was measured by a spectrophotometric method that used a modified dopachrome method. Forty microliters of curcumin solutions at different concentrations were added into each well of a 96-well plate with 140 μL of 2 mg/mL L-DOPA and 20 μL of 10 U/μL tyrosinase. After incubation at room temperature for 30 min, the absorbance was read using an ELISA reader at a wavelength of 475 nm. The percentage of tyrosinase inhibition was calculated as follows:

% tyrosinase inhibition = ((*Ab*_control_ − *Ab*_sample_)/*Ab*_control_) × 100% (1)

### 2.2. Preparation of Gelatin/Chitosan Bilayer Nanofibrous Scaffolds (GC)

#### 2.2.1. Preparation of Chitosan Scaffolds

Chitosan solution with the concentration of 2 wt% was prepared by dissolving the chitosan powder in 2 *v*/*v*% acetic acid under magnetic stirring at 200 rpm for 4 h at room temperature. Planar membranes were performed by pouring five milliliters of the chitosan solution into a 6-cm-diameter dish and frozen at −80 °C for 24 h before lyophilization. The dried chitosan scaffolds were neutralized with 1 N NaOH for 1 h and then copiously rinsed with deionized water. After freeze-dried, the chitosan scaffolds were cross-linked with 50% glutaraldehyde for 45 min.

#### 2.2.2. Preparation of GC/L/C Bilayer Nanofibrous Scaffolds

Gelatin solution with a concentration of 17 wt% was prepared by dissolving 1.53 g gelatin powders in 9 mL formic acid (85%). A clear aqueous solution of polyvinyl alcohol (10 wt% PVA) solution prepared by dissolving 1.0 g PVA in 9 mL deionized water at 70 °C was added to the gelatin solution with a gelatin/PVA ratio of 9/1 *v*/*v*. The solution was agitated at room temperature for 1 h. Four hundred μg/mL curcumin and 625 μg/mL LR extract were added to the gelatin/PVA solution to produce gelatin/PVA/L/C solution. Then the gelatin/PVA solution containing curcumin and LR extract was electrospun onto the chitosan scaffolds to produce the GC/L/C bilayer scaffold. The collection distance was 10 cm. The applied voltage was controlled at 20 kV. The solution flow rate was 0.1 mL/h. After electrospinning, the nanofibers were cross-linked in 50 wt% glutaraldehyde vapor for 45 min. The prepared GC/L/C bilayer nanofibrous scaffolds were sterilized with ultraviolet (UV) radiation for 1h.

### 2.3. Morphological and Physicochemical Characterization of Gelatin/PVA Nanofibers (GEL)

#### 2.3.1. Microscopic Structure Evaluation

The structure of dry GEL nanofibers was observed by using a Field emission scanning electron microscopy (FE-SEM; JEOL JSM-6700F, Tokyo, Japan). The fiber diameters were measured as mean values of 20 measurements in the SEM images and analyzed using Image J.

#### 2.3.2. In Vitro Biocompatibility Assay

The biocompatibility of the membranes was performed on mouse fibroblasts (L929). The electrospun membrane was cut into a circle with a diameter of 1.5 cm in a 24-well plate and sterilized with UV radiation for 1 h. The L929 cells were grown in triplicate with the membrane-conditioned well at a density of 1 × 10^4^ cells/well in a 24-well, flat-bottomed tissue culture plate in triplicate. After 48 h-incubation, the MTT assay was performed to evaluate the cell viability.

#### 2.3.3. Immunofluorescence Staining

Gelatin/PVA solution containing curcumin and LR extract were electrospun on the coverslip. L929 cells were seeded on nanofibers in a density of 1 × 10^4^/well and grown on GEL nanofibers at 37 °C for 48 h. After removing the supernatant and washing with PBS for 3 times, the L929 cells were fixed using methanol: acetone 1:1 for 10 min and soaking with 3% milk for 30 min. For the immunofluorescence staining, the nanofibrous membranes were labeled with anti-*β*-actin antibodies as 1st antibody. After removing the 1st antibody, washed by PBS solution for 3 times and added FITC as the 2nd antibody for 1 h. Then 4′,6-diamidino-2-phenylindole (DAPI) staining for 10 min was performed for quantitative analysis.

### 2.4. In Vivo Wound-Healing Assay

#### 2.4.1. In Vivo Animal Evaluation

Male Sprague–Dawley (SD) rats weighing 350–400 g were used and treated according to the Ethical Guidelines of the Animal Center at China Medical University (CMU) (Taichung, Taiwan). The animal use protocol (protocol ID: CMUIACUC-2017-253) approved by the Institutional Animal Care and Use Committee (IACUC) of CMU on 15 February 2017.

#### 2.4.2. Streptozotocin (STZ)-Induced Diabetic Models in Rats

SD rats weighing 350–400 g were used. On experimental day 1, all rats were fasted for 24 h prior to STZ treatment. The STZ was dissolved in 50 mM sodium citrate and injected at 55 mg/kg. On experiment day 4, the blood glucose of STZ-injected rats was measured to confirm diabetes condition.

#### 2.4.3. Wound-Healing Measurement in STZ Diabetic Rats

The hair (5 × 8 cm^2^) on the back of each rat under anesthesia with ether was removed using a hair remover. After removing the skin of four areas (15 × 15 mm^2^), the wound surfaces were disinfected with I_2_. A GC membrane, GC/L membrane, GC/C membrane, GC/L/C scaffold, a commercial wound dressing (Comfeel^®^), and gauze (control) were placed on the wound surfaces randomly on each rat for 4, 7 and 14 days. The recovery ratio was calculated as (*Ai* − *Au*)/*Ai* × 100%, where Ai is the initial area of the wound, and Au is the area of the unrecovered wound surface.

#### 2.4.4. Histopathological Studies

Skin specimens were cut and immersed in normal 10% buffered formalin for hematoxylin and eosin (HE) staining; the skin specimens were immersed in Bouin’s solution for Masson’s trichrome staining. The fixed skin specimens were processed routinely and viewed under a light microscope to evaluate collagen formation and wound-healing processes.

#### 2.4.5. Collagen Assay in the Wound Area

Skin samples from the wound area were dried and ground into powder. Ten percent pepsin was mixed with the skin sample powder and incubated at 4 °C overnight for protein extraction. A QuickZyme collagen assay kit was applied for collagen quantitation.

### 2.5. Western Blotting Assay

Eighty μg of each sample was mixed with 5X loading dye and heated at 95 °C for 5 min. The upper layer of sodium dodecyl sulfate polyacrylamide gel electrophoresis (SDS-PAGE) is 3.75% Stacking gel, and the lower layer is 12% separating gel. After loading the protein samples, the electrophoresis runs 120 mV electrophoresis and run for 2–3 h. After electrophoresis, the protein was transferred to the methanol activated polyvinylidene difluoride (PVDF) membrane at 100 volts, 4 °C for 2 h. Remove the PVDF membrane and immerse it in a 5% (*w*/*v*) blocking buffer to shake at room temperature for one hour. The PVDF membrane was placed reacted with primary antibody (TGF-*β*) overnight at 4 °C. After washing three times, added the secondary antibody (2 μL/mL 1X Tris-buffered saline (TBS) and put it at 4 °C for 2 h. After removing the PVDF membrane by washing buffer for 3 times, add Chemiluminescence Reagent Plus was added to capture images with a cold light and fluorescent image processing system.

### 2.6. Inflammation Index Assay

The inflammatory index was detected using TNF-*α* and IL-6 detection ELISA kit. One hundred μL of the diluted detection antibody (TNF-*α*, IL-6) was incubated for at least 2 h. Then, 100 μL of the Late Color Development Enzyme was added and incubated for 30 min. After adding 100 μL of TMP solution for 5 min, 100 μL stop solution was added to stop the reaction. The absorbance was measured by ELISA at a wavelength of 450 nm (Molecular Devices, San Jose, CA, USA).

### 2.7. Statistical Analysis

The results are presented as the mean ± standard deviation. Statistical analysis was conducted using Student’s t-test or one-way analysis of variance followed by a post hoc Fisher’s least significant difference test for multiple comparisons. Levels of statistical significance were set at *p* < 0.05.

## 3. Results

### 3.1. Effects of Curcumin and LR Extract on Cell Viability and Biochemical Function

#### 3.1.1. Cytotoxicity of Curcumin against L929 Cells

**Figure 1:**
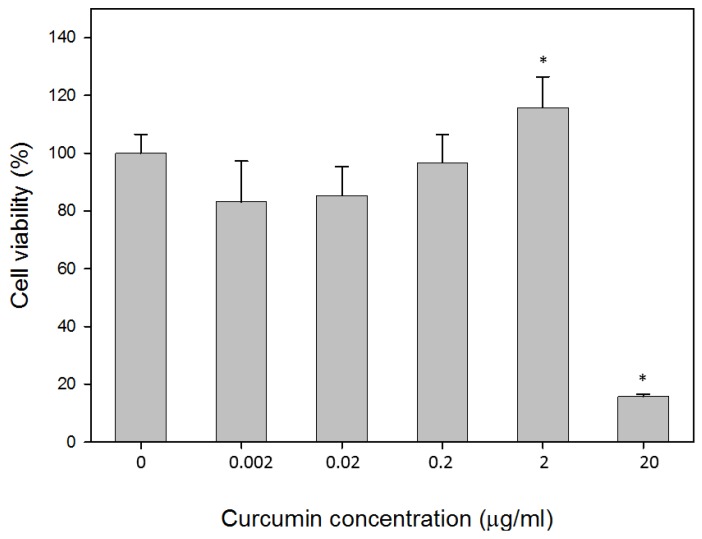
Effects of curcumin on L929 cell viability. Cytotoxicity was determined using MTT assay after 24 h treatment with the indicated concentrations. Values are expressed by mean ± S.D. Asterisk (*) indicates statistically significant differences (*p* <0.05) when compared with the control.

L929 cells incubated with curcumin for 24 h at concentrations of 0.002, 0.02, 0.2, 2 and 20 μg/mL. The viability of the cultures was shown in Figure 1. Following the incubation of L929 cells with 2 μg/mL of curcumin, approximately 15.7% (*p* < 0.05%) increase in cell viability was observed. L929 cultures treated with curcumin at a concentration of 20 μg/mL showed 84.7% inhibition in cell growth.

#### 3.1.2. Effect of Curcumin on an in Vitro Cell Migration Assay

**Figure 2:**
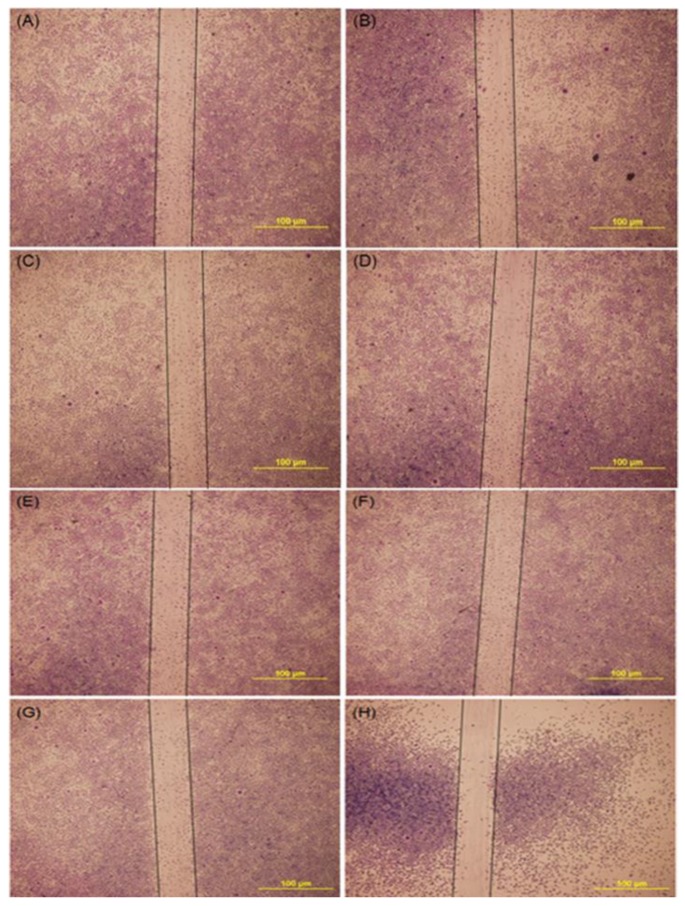
Optical images of L929 cells migration assay treated with various concentration of curcumin. (**A**) control (**B**) 0.00002 μg/mL (**C**) 0.0002 μg/mL (**D**) 0.002 μg/mL (**E**) 0.02 μg/mL (**F**) 0.2 μg/mL (**G**) 2 μg/mL (**H**) 20 μg/mL. Scale bar is 100 μm.

L929 cells were planted in 12-well culture dishes and using a tip to draw a line in the middle of the culture dish. Cultures treated with various concentrations of curcumin were observed after 48 h incubation. Figure 2 shows a low level of curcumin, less than 2 μg/mL, obtained similar results to the control (Figure 2A–G); the higher level of curcumin (20 μg/mL) reduced the cell migration. The result showed that curcumin at the range of 0–2 μg/mL had no apparent influence on the migration kinematics of fibroblasts to the wound area (scratch line).

#### 3.1.3. Inhibition Effect of Curcumin on Tyrosinase Activity

**Figure 3:**
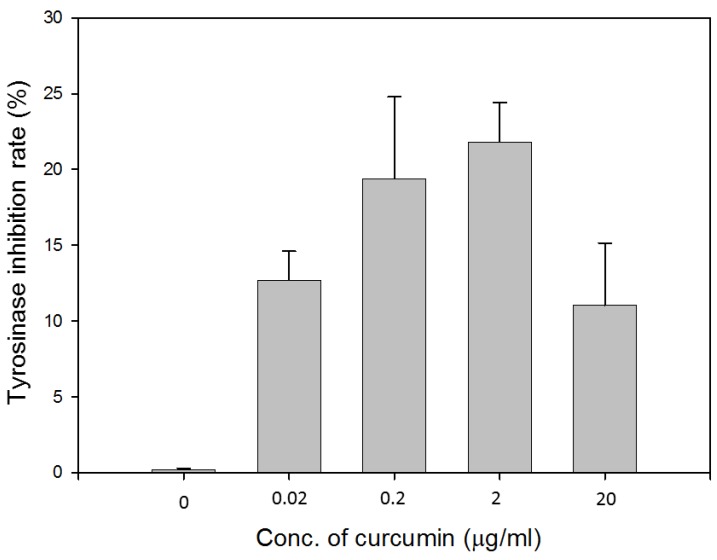
Inhibitory effects of various concentrations of curcumin against tyrosinase activity.

Tyrosinase inhibitory activity was determined by a spectrophotometric method using 2 mg/mL L-DOPA as the substrate. As shown in Figure 3, curcumin at the concentrations of 0.2 and 2 μg/mL inhibited tyrosinase activity by 19.4% and 21.8%, respectively. Curcumin might play a role in preventing excess melanin produced during the wound healing process. Based on the results from the cell viability assay, cell migration assay, and tyrosinase inhibition assay, curcumin at the concentration of 2 μg/mL was used for further experiments.

#### 3.1.4. The Drug Loading Concentration of Curcumin and LR Extract

In our previous study, the LR extract at a concentration of 3.12–6.25 μg/mL, accelerated the cell viability of L929 fibroblasts and demonstrated antimicrobial activity [16]. Curcumin at the concentration of 2 μg/mL promoted cell viability and biochemical function. Based on the drug released efficiency, gelatin nanofibers containing drugs at 200-fold could release an effective dosage of drugs [11]. Thus, 625 μg/mL LR extract and 400 μg/mL curcumin were added to electrospun gelatin nanofibers.

### 3.2. Morphological and Physicochemical Characterization of GEL Nanofibers

#### 3.2.1. Microscopic Structure Evaluation of GEL Nanofibers

**Figure 4:**
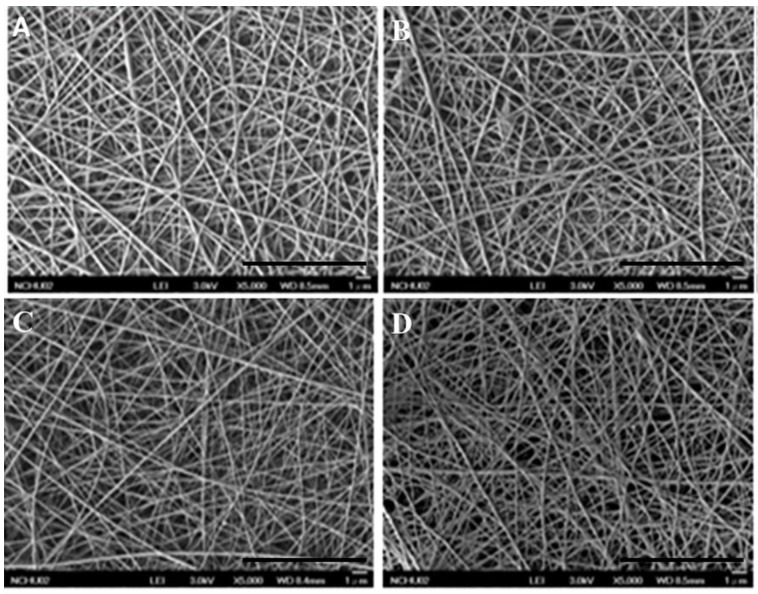
Scanning electron microscopy (SEM) images of various GEL nanofibers. (**A**) GEL, (**B**) GEL/L, (**C**) GEL/C, (**D**) GEL/L/C. Bars: 10 μm.

The various gelatin nanofibers electrospun at 0.1 mL/h at 20 kV had filamentous and highly porous structure. The SEM images of nanofibers were observed by SEM (Figure 4A–D) and appeared smooth and homogeneous. The average diameters of GEL, GEL/L, GEL/C, and GEL/L/C are 139.6 ± 59.16, 125.2 ± 35.87, 72.66 ± 21.52 and 102.8 ± 36.01 nm respectively. All four nanofibers are close to 100 nm, GEL/C, and GEL/L/C especially.

#### 3.2.2. Biocompatibility of Various GEL Nanofibers.

**Figure 5:**
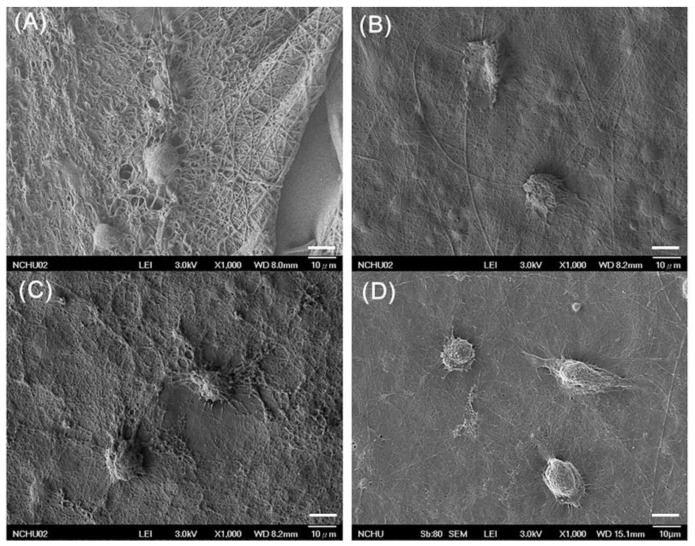
SEM images of L929 cells co-cultured with different GEL nanofibrous membranes for 24 h. (**A**) GEL (**B**) GEL/L (**C**) GEL/C (**D**) GEL/L/C. Bars: 10 μm.

**Figure 6:**
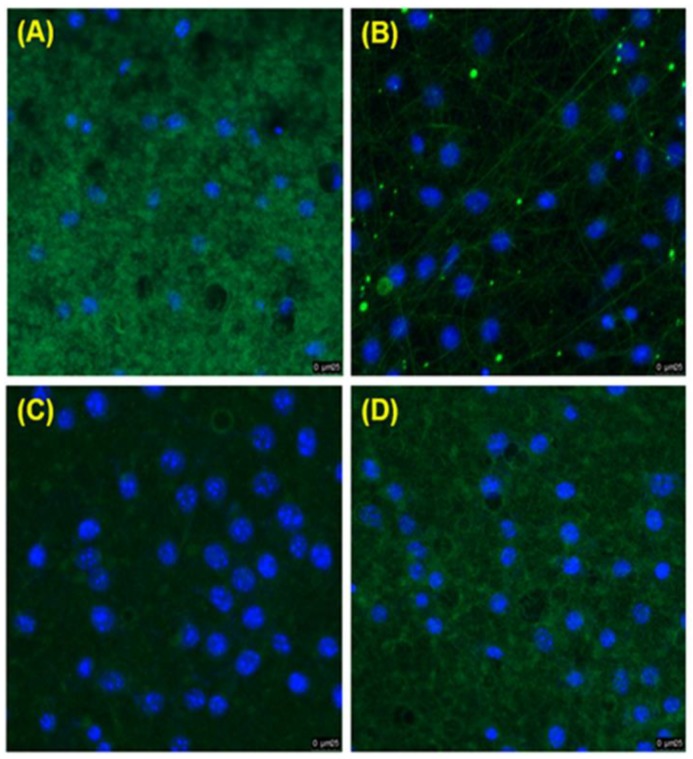
Immunofluorescence staining of L929 cells seeded and grown on different GEL membranes 24 h. (**A**) GEL (**B**) GEL/L (**C**) GEL/C (**D**) GEL/L/C.

L929 cells were co-cultured on GEL, GEL/L, GEL/C, and GEL/L/C nanofibrous membrane for 24 h. Then SEM analysis was performed to evaluate the biocompatibility of membranes (Figure 5). In Figure 5A–D, L929 cells were nicely spread on all GEL nanofibers. The GEL membranes, with or without the addition of the drug, created a beneficial environment for cell attachment. For the immunofluorescence assay (Figure 6), the GEL nanofibers (green) were reacted with anti-*β*-actin antibodies and labeled with goat anti-mouse FITC antibodies (Figure 6A–D). The result of immunofluorescence assay also demonstrated that GEL, GEL/L, GEL/C, and GEL/L/C nanofibers are biocompatible to L929 cells.

### 3.3. Wound Healing Effect of Bilayer Nanofibrous Scaffolds in STZ Diabetic Rats

#### 3.3.1. GC/L/C Bilayer Nanofibrous Scaffolds Accelerated Wound Recovery Rate

**Figure 7:**
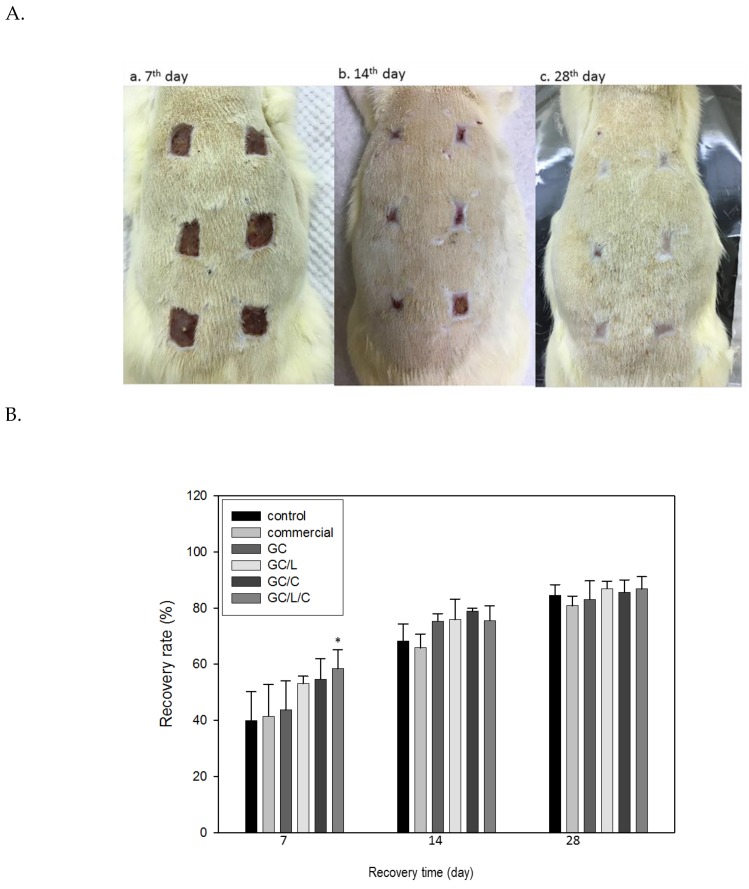
(**A**) Photographs of the wound areas that were treated with a GC, GC/L, GC/C, and GC/L/C scaffolds, commercial (Comfeel^®^) or gauze (control) at seventh day (**a**), 14th day (**b**) and 28th day (**c**). The arrangements of various membranes’ positions on the rats were changed randomly. (**B**) The recovery rates of wounds with various treatments. The surface area changes of the wound were monitored from four animals in each group. *Compared with the control group, *p* < 0.05.

Figure 7A shows the appearances of the wound areas that were treated with GC, GC/L, GC/C and GC/L/C scaffolds, commercial (Comfeel^®^) or gauze (control) at seventh day (a), 14th day (b) and 28th day (c) in STZ-induced diabetic rats. On the seventh day, the recovery rates of diabetic rats treated with GC, GC/L, GC/C and GC/L/C scaffold, Comfeel^®^ or gauze (control) were 43 ± 10%, 53 ± 3%, 54 ± 12%, and 58 ± 7% (*p* < 0.05), 41 ± 11% and 40 ± 16%, respectively (Figure 7B). Treatment with the GC/L/C scaffold on the seventh day achieved the best performance, with a 58 ± 7% increase in recovery rate when compared to that of the control. On the 14th day, the recovery rates of diabetic rats treated with GC, GC/L, GC/C, and GC/L/C scaffold, commercial (Comfeel^®^) or gauze (control) were 75 ± 3%, 76 ± 7% and 79 ± 1%, 76 ± 5%, 66 ± 5%, and 68 ± 6%, respectively. Treatments with GC, GC/L, GC/C, and GC/L/C bilayer scaffold showed a higher recovery rate than the control group. On the 28th day, the recovery rates of diabetic rats treated with GC, GC/L, GC/C, and GC/L/C scaffold, Comfeel^®^ or gauze (control) were 83 ± 7%, 87 ± 3%, 86 ± 4%, and 87 ± 4%, 81 ± 3% and 85 ± 4% respectively. The recovery rates of all treatments are higher than 80%, with no visible difference between them.

#### 3.3.2. GC/L/C Bilayer Scaffold Increased Collagen Secretion in the Wound Area

**Figure 8:**
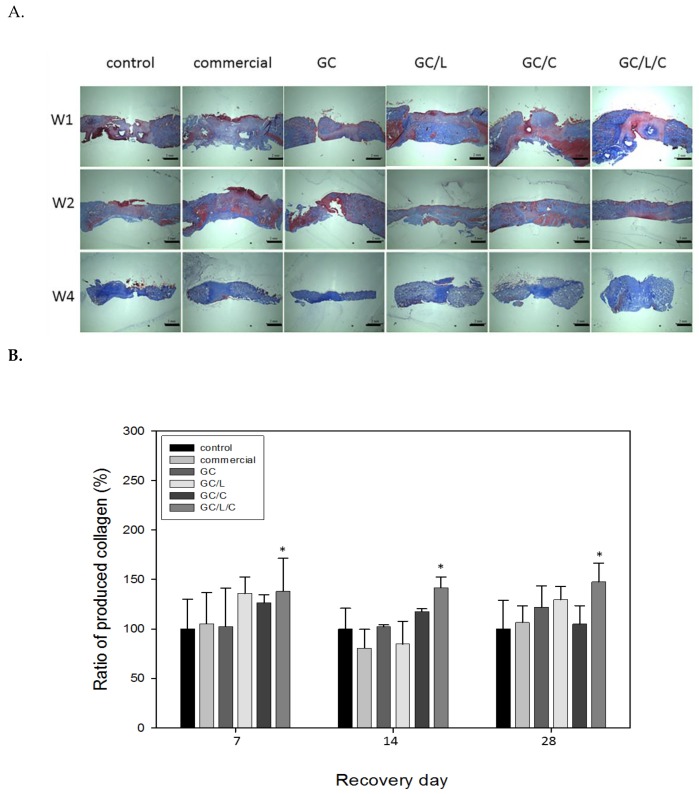
(**A**) Masson’s trichrome staining images of wound area treated with a GC, GC/L, GC/C, and GC/L/C scaffolds, commercial (Comfeel^®^) or gauze (control) at seventh, 14th and 28th day. (**B**) The collagen content of the seventh, 14th and 28th day-specimens of wound areas treated with different scaffolds. * Compared with the control group, *p* < 0.05.

In Figure 8A, the skin specimens treated with various wound dressing at the seventh, 14th and 28th day (Figure 8A) are shown. The collagen fibers were stained in blue with Masson’s trichrome staining. Figure 8B demonstrates that the wounds treated with the GC/L/C scaffold produced higher collagen levels than those treated with the other wound dressings at 7, 14, and 28 days. Compared with the control, the GC/L/C scaffold increased collagen content by 38.03% (*p* < 0.05), 41.59% (*p* < 0.05), and 47.58% (*p* < 0.05) in the wound on the seventh, 14th and 28th days.

#### 3.3.3. In Vivo Treatments with GC/C and GC/L/C Bilayer Scaffold Increased TGF-*®* Protein Level in Wounds

**Figure 9:**
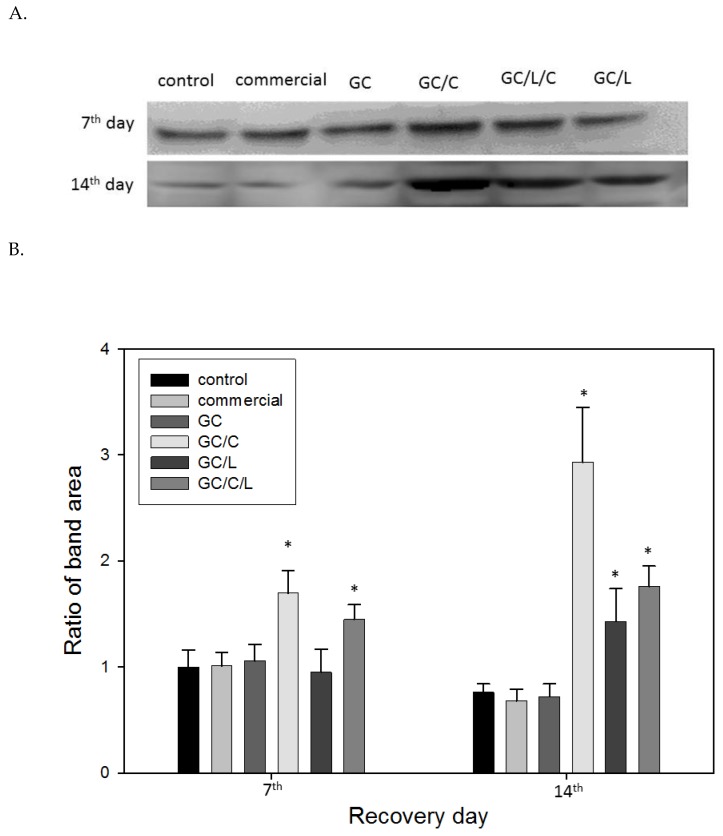
(**A**) Western blot analysis of TGF-*β* protein in wounds treated with a GC, GC/L, GC/C, and GC/L/C scaffolds, commercial (Comfeel^®^), or gauze (control) at seventh and 14th day. (**B**) TGF-*β* protein band-ratio in wounds treated with various membranes on the 7th and 14th day.

Figure 9A presents the Western blot analysis of TGF-*β* protein in wounds with various treatments. The ratio of TGF-*β* protein band areas are shown in Figure 9B. In comparison with the control, the TGF-*β* protein increased by 70.3% (*p* < 0.05%) and 40.15% (*p* < 0.05%) with GC/C and GC/L/C treatment, respectively, on seventh day. On the 14th day, the TGF-*β* protein increased by 189.7% (*p* < 0.05%), 59.3% (*p* < 0.05%) and 107.5% with GC/C, GC/L and GC/L/C treatment, respectively. The TGF-*β* protein contents show no obvious difference in wounds treated with commercial dressing and GC nanofibers at seventh and 14th days.

#### 3.3.4. In Vivo Treatments with GC/L/C Nanofibers Reduced Pro-Inflammatory Cytokines IL-6 and TNF-*α* Protein Level in Wounds

**Figure 10:**
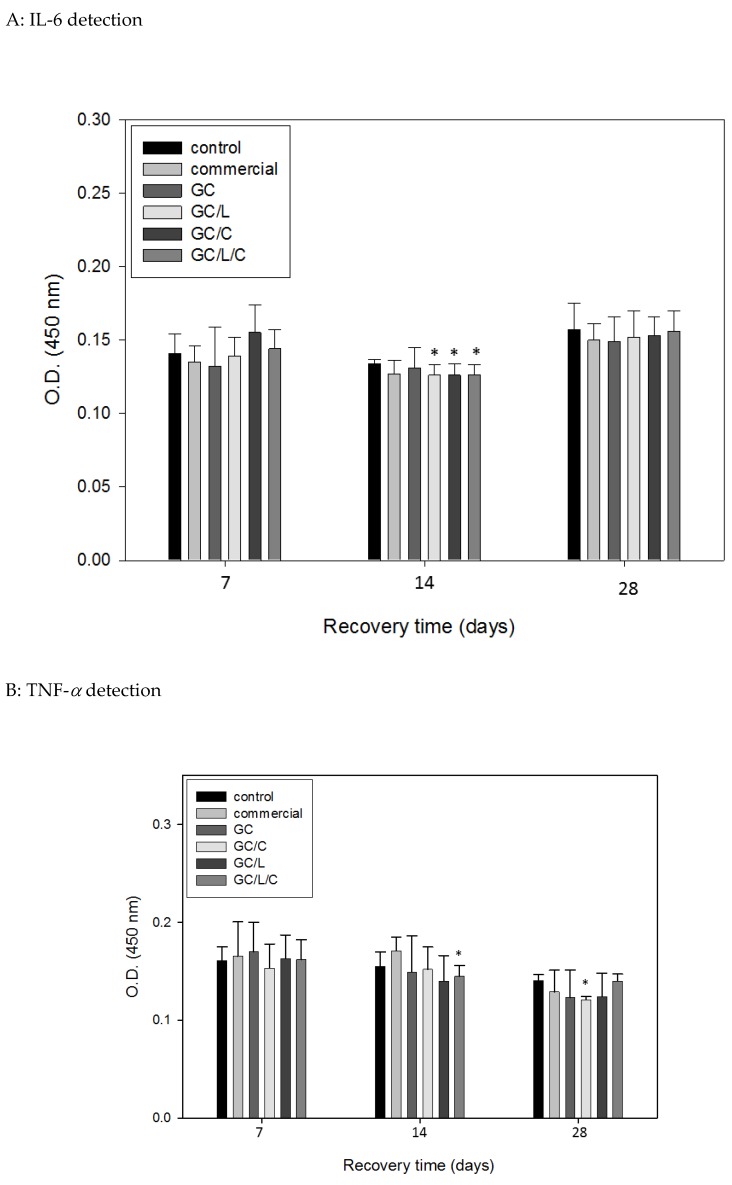
Comparisons of IL-6 (**A**) and TNF-*α* (**B**) level in diabetic rats treated with a GC, GC/L, GC/C, and GC/L/C scaffolds, commercial (Comfeel^®^) or gauze (control) at seventh, 14th and 28th day.

We show the wounds treated with GC/C, GC/L, and GC/L/C reduced pro-inflammatory cytokines IL-6 level (*p* < 0.05) on the 14th day in Figure 10A. The TNF-*α* level in the specimen treated with GC/L/C was reduced by 6.45% (*p* < 0.05) on the 14th day, in the specimen treated with GC/L, it was reduced by 13.6% (*p* < 0.05) on the 28th day (Figure 10B).

## 4. Discussion

Wound healing is a complex process that re-establishes the integrity of the damaged tissue. In this study, we successfully utilized the pharmaceutical effects of curcumin and LR extract within bilayer nanofibrous scaffolds to improve wound healing in an STZ diabetic rat model.

When L-929 fibroblasts were incubated with 2 μg/mL curcumin extract for 24 h, the cell viability increased by up to 15.7% (Figure 1). Curcumin at the concentration of 2 μg/mL was capable of reducing 21.8% of tyrosinase activity. It has been reported that a low level of curcumin between 1–5 μg/mL demonstrated no-cytotoxicity [24,25]. Various studies have shown the infiltration of fibroblasts into wound sites when treated with curcumin in a rat model [26,27,28]. The influence of curcumin on cell migration is based on the dosage and cell lines. The high concentration of curcumin (9.2 μg/mL) suppressed cell proliferation and migration in cardiac fibroblasts [29]. Highly migratory cells showed a decrease in migration speed and directionality when treated with 2 or 5 μM (0.74–1.84 μg/mL) of curcumin [30]. In this study, we tried to explore a potential mechanism of curcumin in wound healing by performing the scratch assay. The results showed that curcumin at the concentration of the range of 0–2 μg/mL gave no noticeable effect when compared with the control group. A similar result has been reported, where 4.07 μg/mL did not influence keratinocyte migration [31].

In order to mimic the structure of the extracellular matrix (ECM), it is appropriate to produce fibers with diameters thinner than 100 nm [32]. The various gelatin nanofibers electrospun at 0.1 mL/h at 20 kV presented filamentous and highly porous structures. The average diameters of GEL, GEL/L, GEL/C, and GEL/L/C are 139.6 ± 59.16, 125.2 ± 35.87, 72.66 ± 21.52, and 102.8 ± 36.01 nm, respectively (Figure 4). All four nanofibers are close to 100 nm, GEL/C and GEL/L/C especially. The characteristics of the nanofibers were investigated in our previous study. The water-retention rate of gelatin nanofibers increased by only 1.5- to 1.73-fold after 3–48 h of soaking. The weight losses ranged from 30% to 42.5% after soaking in water for 1–21 days. The contact angles were 44 ± 4° for the electrospun gelatin membranes [11]. Evidence of immunofluorescence staining further supports that GEL nanofibers with the addition of curcumin and LR extracts are biocompatible to L929 cells (Figure 6A–D). Chitosan scaffolds obtain many advantages, like accelerating tissue regeneration, biocompatible, and antibacterial property [6,7,8]. The addition of GEL nanofibers onto the chitosan scaffolds to produce the bilayer nanofibrous scaffold could prevent curled deformation of the nanofibers and promote cell adhesion, migration, differentiation, and proliferation [33,34,35].

The wound healing effects of GC, GC/L, GC/C, and GC/L/C bilayer nanofibrous scaffolds were evaluated by incision on STZ-induced diabetic rats. The recovery rates of healthy rats and diabetic rats were about 85% and 45% of the healthy and diabetic rats on the seventh day, respectively. The STZ-induced diabetic rats showed a delayed wound healing process. In order to reduce other influences on the recovery rate, the positions of different treatments on the rats were arranged randomly in each group. Compared with the other treatments (control, commercial, GC, GC/L, and GC/C), the wound area treated with GC/L/C nanofibers showed the highest recovery rate (58.7%, *p* < 0.05%) on the seventh day (Figure 7B). On the 14th day, the recovery rates of control (gauze), GC, GC/L, GC/C, and GC/L/C treatments were 68 ± 7%, 75 ± 3%, 76 ± 7% and 79 ± 1%, 76 ± 5%, respectively. GC membranes, with or without drug addition, all showed a higher recovery rate than the control group. On the 28th day, the recovery rates of diabetic rats with various treatments were higher than 80%, with no apparent difference between all treatments.

Assessment of collagen content in wound tissues of control and other treated wounds showed a significant increase in the amount of total collagen observed in GC/L/C-treated wounds (Figure 8). There was no difference in the content of Type I collagen (data not shown). It has been reported that more type III collagen is synthesized than type I collagen [36]. Type III collagen is associated with an early increase in the proliferation phase, which may function in providing wound structure and support for further wound healing. In the tissue remodeling phase, the last stage of wound healing, collagen type III is replaced by the stronger type I collagen [37,38,39]. Compared with the control group, the wound area treated with GC/L/C obtained 41.6%, 38%, and 47.6% increase of total collagen on the seventh, 14th, and 28th days, respectively. It might suggest that a GC/L/C bilayer scaffold increases collagen synthesis and function in providing wound structure and support for further wound healing.

TGF-*β* is released in wound areas following tissue damage through platelet degranulation, which plays a crucial role in the process of wound healing. It has been reported that curcumin and shikonin (the active principle in the root of Lithospermum) promote TGF-*β* release in wounds [17,40,41]. The wounds treated with GC/C, GC/L, and GC/L/C bilayer scaffolds obtained a higher level of TGF-*β* release on the 14th day in the Western blotting analysis (Figure 9).

Pro-inflammatory cytokines, IL-6 and TNF-*α*, might function as inflammatory biomarkers. These results could provide evidence for the anti-inflammatory effects of the addition of curcumin and LR extract within GC nanofibers. Evidence showed that the levels of IL-6 and TNF-*α* in STZ diabetic rats in human skin wounds. Compared with the control, GC/C, GC/L, and GC/L/C bilayer scaffold treatments decreased the IL-6 level on the 14th day (*p* < 0.05%) (Figure 10A), GC/L/C bilayer scaffold treatments decreased the TNF-*α* level on the 14th day (*p* < 0.05%) (Figure 10B). These results could provide evidence for the anti-inflammatory effects of the addition of curcumin and LR extract within the GC bilayer nanofibrous scaffold. It has been reported that the levels of IL-6 and TNF-*α* in STZ diabetic rats are higher than healthy rats [42]. It might explain that no apparent effect of GC/C, GC/L and GC/L/C treatments was observed within the first week.

Curcumin and LR extracts performed different effects in wound treatments. According to the results of the Western blotting assay and histopathological studies, GC/L treatment obtained better performance in increasing collagen synthesis (Figure 8), and GC/C treatment showed a higher level of TGF-*β* protein secretion (Figure 9). The treatment of incision wounds in STZ diabetic rats with GC/L/C bilayer membranes demonstrated a potent curative activity, which might be attributed to the synergistic effect of the addition of curcumin and LR extract.

## 5. Conclusions

Gelatin nanofibers containing curcumin and LR extract were electrospun onto chitosan scaffolds to manufacture bilayer nanofibrous scaffolds with a healing effect. The benefits of the highly porous GC/L/C bilayer scaffold could reduce the frequency of dressing changes and minimize discomfort. In an STZ diabetic rat model, the GC/L/C bilayer scaffold out-performed the control groups in terms of collagen synthesis, TGF-*β* production, anti-inflammatory effect, and promoted the wound healing process. Based on their wound healing activity, the GC/L/C bilayer nanofibrous scaffold could be an excellent candidate for wound dressing.
